# Rumen perforation caused by horn injury in two cows

**DOI:** 10.1186/s13028-016-0185-8

**Published:** 2016-01-20

**Authors:** Ueli Braun, Christian Gerspach, Manuela Stettler, Daniela Grob, Titus Sydler

**Affiliations:** 1 Department of Farm Animals, Vetsuisse-Faculty, University of Zurich, Winterthurerstrasse 260, 8057 Zurich, Switzerland; 2 Vetsuisse-Faculty, Institute of Veterinary Pathology, University of Zurich, Winterthurerstrasse 260, 8057 Zurich, Switzerland

**Keywords:** Ultrasonography, Cattle, Rumen, Perforation, Trauma, Horn injury, Peritonitis

## Abstract

Post-operative complications of trocarisation and rumenotomy are the most common causes of peritonitis associated with a rumen disorder. Since horn injury leading to rumen perforation has not previously been reported in the literature, two cows with this condition are reported. Small superficial skin lesions were observed in one of the cows and the other had a perforating skin lesion in the left abdomen. Both cows had signs of hypovolaemic shock. Ultrasonography revealed hypoechoic fluid, echoic lesions and occasional fibrinous septa caudoventral to the reticulum. Caudally the fluid extended to the left flank fold and occupied about one third of the peritoneal cavity. The area of the skin perforation in the left abdomen was swollen and the muscle layers could not be differentiated using ultrasonography. Diffuse fibrino-purulent peritonitis was diagnosed in both cows, and because of a poor prognosis, they were euthanased and necropsied. Perforation of the abdominal wall and rumen with diffuse fibrino-purulent peritonitis was present. Ultrasonography is a suitable tool to characterise the inflammatory lesions between the rumen and left abdominal wall and objectify the interpretation of clinical findings. Horn injury should be included in the rule outs for cattle with left abdominal skin wounds and diffuse peritonitis.

## Background

Disorders of the rumen are rarely associated with generalised or severe peritonitis in cattle. Cases are usually characterized by suppurative inflammation between the rumen and serosal surface of the left abdominal wall, or occasionally an empyema, which in severe cases can extend from the diaphragm to the pelvic inlet [1]. The most evident clinical signs are associated with localised or sometimes generalised peritonitis [1–4] and include decreased appetite and milk production, recurrent tympany, diarrhea or constipation, arched back, weight loss and decreased rumen motility. Diagnosis can be confirmed by blind abdominocentesis at a site in the left paralumbar fossa or in a caudal intercostal space [1]; this allows the escape of foul-smelling gas and malodorous watery to viscous exudate. Before the introduction of ultrasonography into veterinary medicine, an exploratory laparotomy was the only method to determine the extent of the lesions in vivo. Today, ultrasonography is the method of choice for evaluation of peritonitis and for guiding collection of fluid via abdominocentesis [5]. Trocarisation and rumenotomy are the most common causes of peritonitis associated with a rumen disorder [1, 2]. In rare cases, transmural necrosis associated with ruminitis can lead to perforation of the rumen wall. Horn injuries are uncommon because the majority of cows kept in freestall operations have been dehorned; in Switzerland, more than 90 % of dairy cows in freestall operations have been dehorned. Nevertheless, horned cows pose a significant risk of injury to other cows. As horn injury causing rumen perforation has not been reported yet, the goal of this study was to describe the clinical, ultrasonographic and pathological findings in two Brown Swiss cows with this type of injury. Both cows were referred to the Department of Farm Animals, University of Zurich, for examination.

## Case presentation

Cow 1 was a six-year- old Brown Swiss cow from a freestall operation with 25 horned cows. The cow had calved unassisted 6 weeks before referral and had incurred a horn injury to her udder and left lateral abdominal wall from another cow 2 weeks before referral. Bloody milk was observed at milking, and anorexia, groaning and ruminal atony were noted 2 days before referral. At the time of admission to the clinic, the general health of the cow was markedly disturbed and anorexia and frequent bruxism were observed. There was enophthalmus, congestion of the scleral blood vessels and a decrease in skin turgor and skin temperature. The heart rate was markedly increased (104 bpm), and the rectal temperature was decreased (37.9 °C). There was a distinct decrease in ruminal contractions, the rumen was fuller than normal and its contents were hard. The withers pinch test elicited grunting, and abdominal guarding was present. Intestinal motility was decreased, and only a small amount of dry faeces was present in the rectum. Multiple, small, superficial skin wounds were observed on both sides of the body.

Cow 2 was an eight-year-old Brown Swiss cow from a freestall operation with 30 horned cows. The cow had calved 8 weeks before referral. The owner noticed a decrease in appetite several days before referral as well as superficial skin lesions on the left side of the body, which were thought to be due to a horn injury from another cow. Clinical examination at the time of admission revealed anorexia and severely disturbed general health. The cow had tachycardia (104 bpm) and a decreased rectal temperature (37.5 °C). There was enophthalmus, congestion of the scleral blood vessels and a decrease in skin turgor and skin temperature. The rumen was fuller than normal and atonic, intestinal motility and the amount of faeces in the rectum were decreased and abdominal guarding was present. Transrectal palpation was difficult because of the increased size of the rumen and its hard contents. A perforating wound with a diameter of 0.5 cm was present in the 12th intercostal space at the level of the mid-thorax on the left side. There was swelling of the skin and mild subcutaneous emphysema in the region of the wound.

Haemoconcentration, leukopenia with a left shift, hypokalaemia, hypophosphataemia and mild metabolic acidosis were seen in both cows (Table 1). Other findings included hypoproteinaemia (cow 1), hyponatraemia (cow 2) and hypocalcaemia (cow 1). A sample of rumen fluid collected with a stomach tube had a normal colour, odour and chloride concentration (cow 1, 26 mmol/l; cow 2, 19 mmol/l), an increased pH (cow 1, pH of 9; cow 2, pH of 8) and increased time (>6 min) for methylene blue reduction testing in both cows.

**Table 1:** Laboratory findings on the day of admission in two cows with rumen perforation caused by a horn injury

Variable	Cow 1	Cow 2	Normal range
Haematocrit (%)	49	45	30–35
Total leukocyte count (×10^3^/µl)	2.5	4.1	5.0–10.0
Total protein (g/l)	52	64	60–80
Fibrinogen (g/l)	2	4	4–7
Urea (mmol/l)	7.2	3.9	2.4–6.5
ASAT (U/l)	121	65	20–103
γ-GT (U/l)	13	26	9–30
Sodium (mmol/l)	141	137	145–155
Potassium (mmol/l)	3.0	2.5	4–5
Chloride (mmol/l)	94	98	96–105
Calcium (mmol/l)	1.76	4.47*	2.3–2.6
Inorg. phosphorus (mmol/l)	1.23	1.02	1.3–2.4
Magnesium (mmol/l)	0.82	1.97*	0.8–1.0
Rumen chloride (mmol/l)	26	19	15–30

* The cow had been treated with 500 ml of a calcium borogluconate solution containing magnesium hypophosphite administered intravenously by the referring veterinarian a few hours before admission to the clinic

**Fig. 1:**
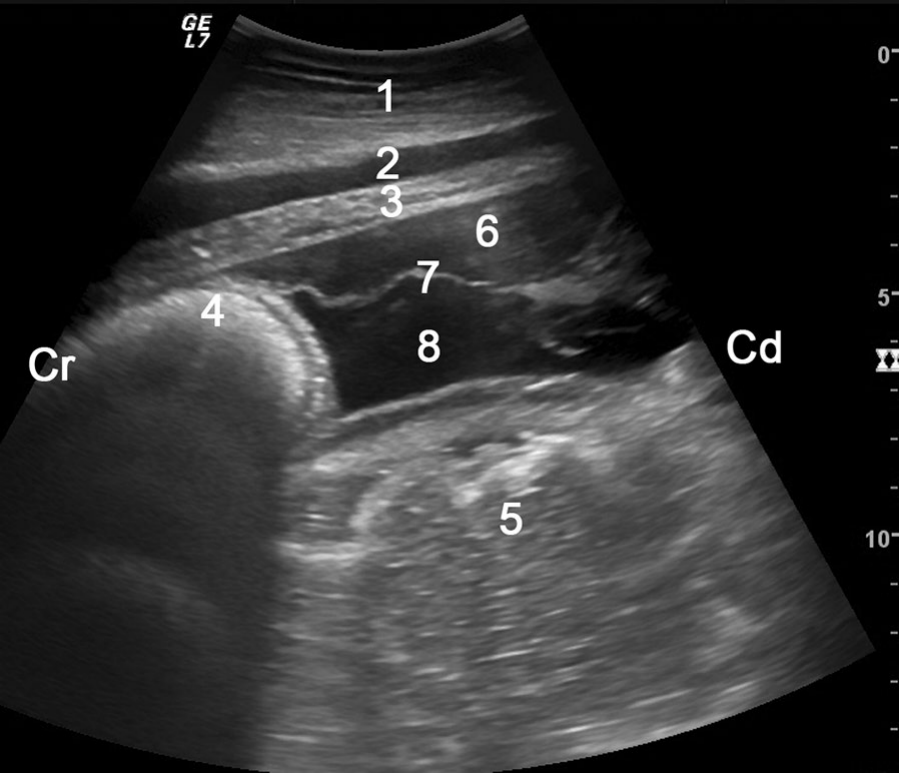
Ultrasonogram reticulum. Ultrasonogram showing peritonitis caudal to the reticulum in cow 2. The view was obtained from the sternal area using a 5.0-MHz convex transducer. The* three layers* of the reticular wall (tunica serosa, tunica muscularis, tunica mucosa) are visible because of fluid accumulation. The abomasum is dilated and one echoic abomasal fold is seen. Hypoechoic fluid with a fibrin strand is evident caudal to the reticulum and ventral to the abomasum. *1* ventral abdominal wall, *2* musculophrenic vein, *3* diaphragm, *4* reticulum, *5* abomasum, *6* echoic cell-rich fluid ventrally, *7* fibrin strand, *8* hypoechoic cell-poor fluid, *Cr* cranial, *Cd* caudal

**Fig. 2:**
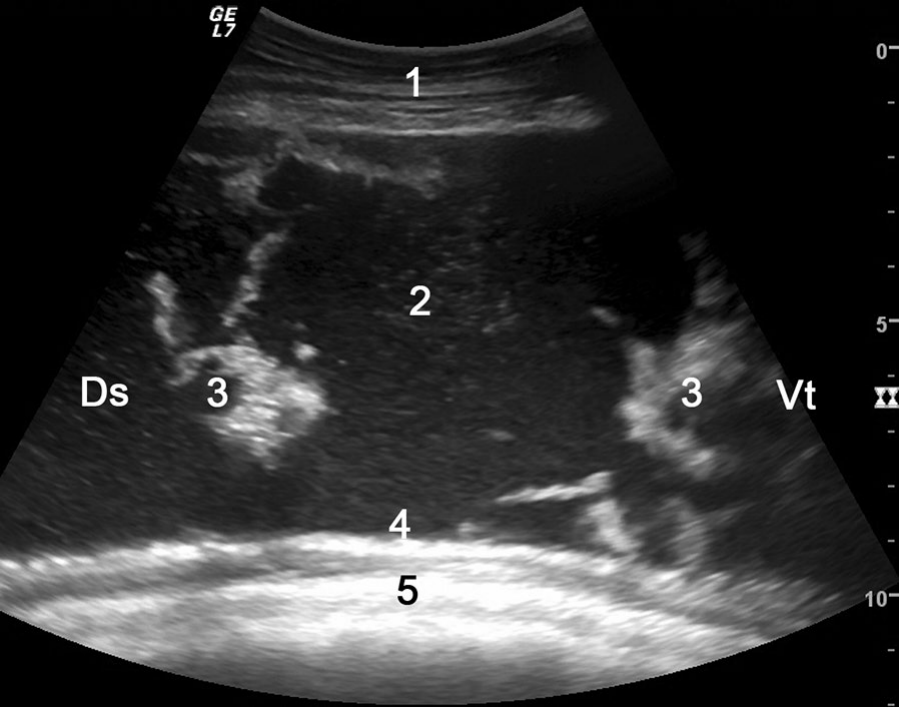
Ultrasonogram showing peritonitis between the rumen and left abdominal wall. Ultrasonogram showing peritonitis between the rumen and left abdominal wall in cow 2. The view was obtained using a 5.0-MHz convex transducer placed in the 12th intercostal space* (lower third*) on the* left side*. A large amount of hypoechoic fluid containing echoic fibrin is evident between the rumen and left abdominal wall. *1* abdominal wall, *2* fluid accumulation, *3* fibrin, *4* greater omentum, *5* rumen wall, *Ds* dorsal, *Vt* ventral

**Fig. 3:**
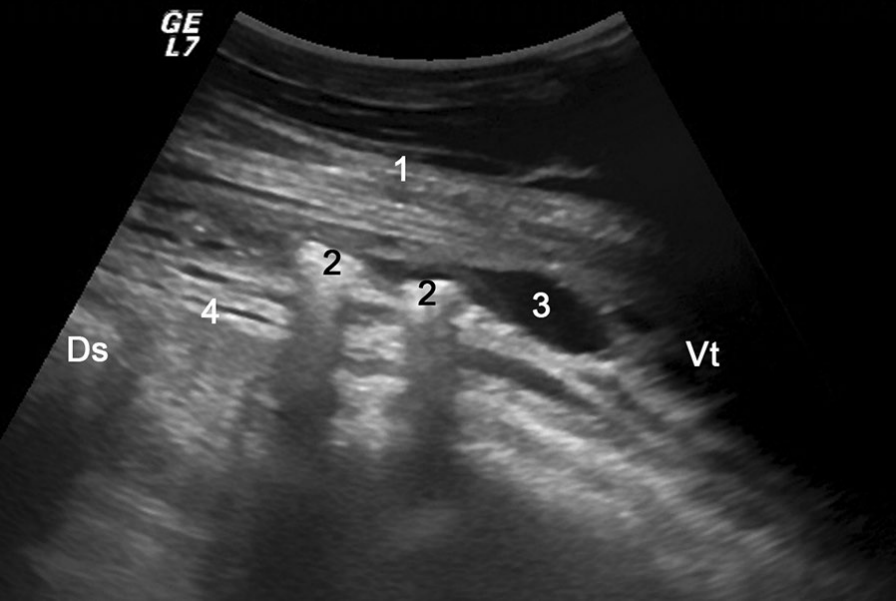
Ultrasonogram of the abdominal wall in the area of skin perforation. Ultrasonogram showing the abdominal wall in the area of a skin perforation in cow 2. A 5.0-MHz convex transducer was used, and the muscle layers and abdominal wall cannot be differentiated because of trauma-induced changes. Gas inclusions and fluid also are apparent. *1* abdominal wall, *2* gas inclusions, *3* fluid, *4* rumen wall, *Ds* dorsal, *Vt* ventral

**Fig. 4:**
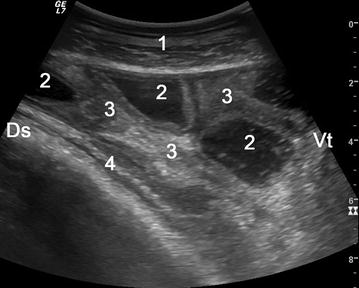
Ultrasonogram in the right flank showing peritonitis. Ultrasonogram obtained using a 5.0-MHz convex transducer placed in the ventral right flank region in cow 2. The intestines are mildly dilated, have a thickened wall and contain fluid. Echoic fibrin is evident between loops of small intestines. *1* abdominal wall, *2* small intestines with thickened wall, *3* fibrin between loops of small intestines, *4* rumen, *Ds* dorsal, *Vt* ventral

Ultrasonographic examination of cow 1 revealed a marked decrease in reticular motility and hypoechoic fluid with a heterogeneous appearance and echoic fibrin caudoventral to the reticulum. Caudally, the fluid extended to the left flankfold and occupied the bottom third of the abdominal cavity. There was atony, mild dilatation and thickening of the wall of the small intestines, and fibrin was observed between loops of intestines. Ultrasonographic examination of cow 2 also showed a marked decrease in reticular motility, and hypoechoic fluid with a heterogeneous appearance and echoic fibrin caudoventral to the reticulum (Fig. 1). The fluid extended to the left flankfold caudally and occupied the bottom third of the abdominal cavity (Fig. 2). Ultrasonography showed that the thickness of the skin was 2.8 cm cranial to the perforation and 3.3 cm in the area of the perforation in cow 2. The skin and muscle layers could easily be differentiated in unaffected areas, but diffuse changes were seen around the wound and the individual muscle layers could not be differentiated (Fig. 3). Emphysema and fluid accumulation were also present. The ultrasonographic appearance of the small intestines was similar to that of cow 1 with atony and thickening of the intestinal wall (Fig. 4). Abdominocentesis in cow 2 yielded yellowish-green, turbid, odourless fluid with a specific gravity of 1.038 and a protein concentration of 55 g/l. Radiographs of the reticulum did not show a reticular foreign body in either cow.

**Fig. 5:**
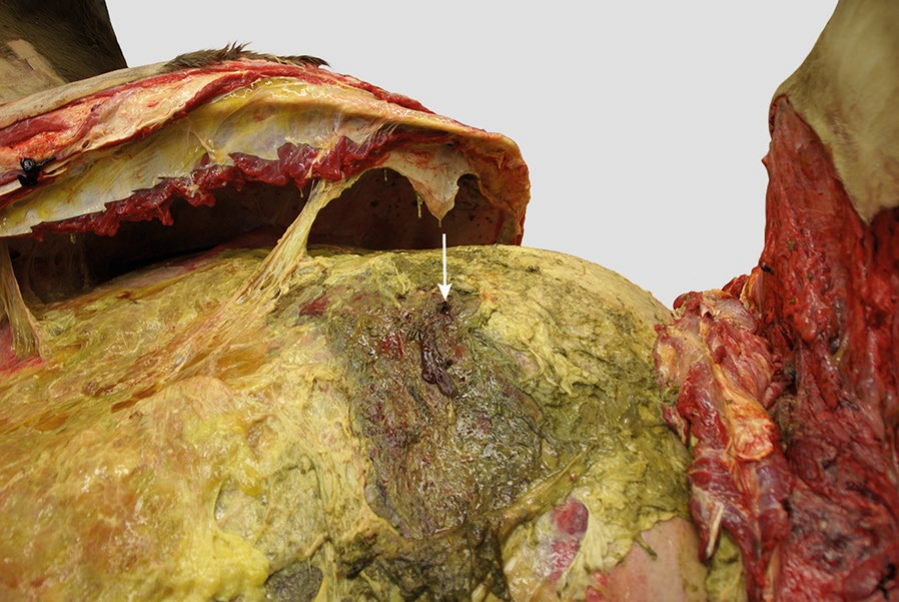
Surface of the rumen with a perforating horn injury. Surface of the rumen in cow 1 with a perforating horn injury (*arrow*). The rumen wall surrounding the perforation is covered with feed particles, and suppurative fibrinous adhesions are seen cranially

**Fig. 6:**
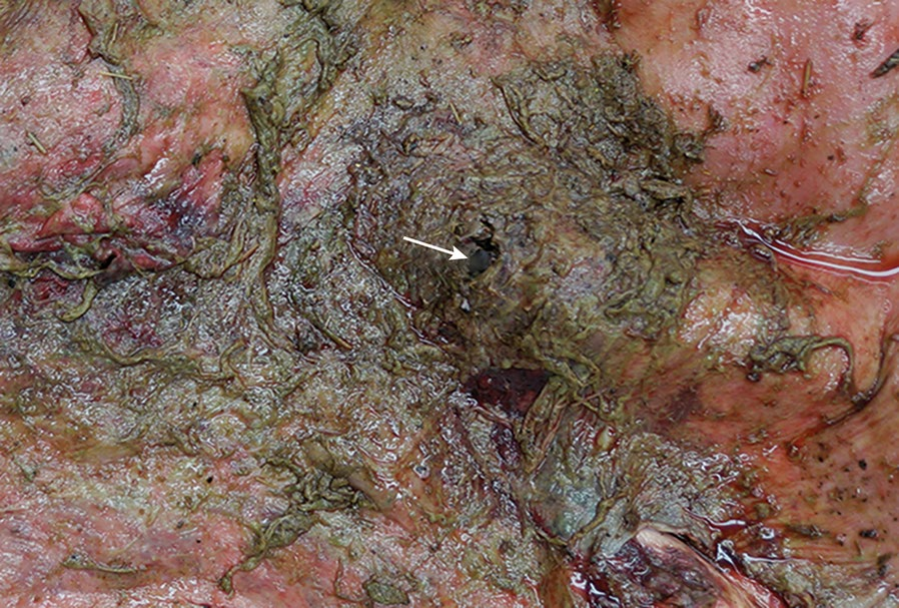
Surface of the rumen with a perforating horn injury. Surface of the rumen in cow 2 with a perforating horn injury (*arrow*). The surface is markedly reddened and covered with feed particles

**Fig. 7:**
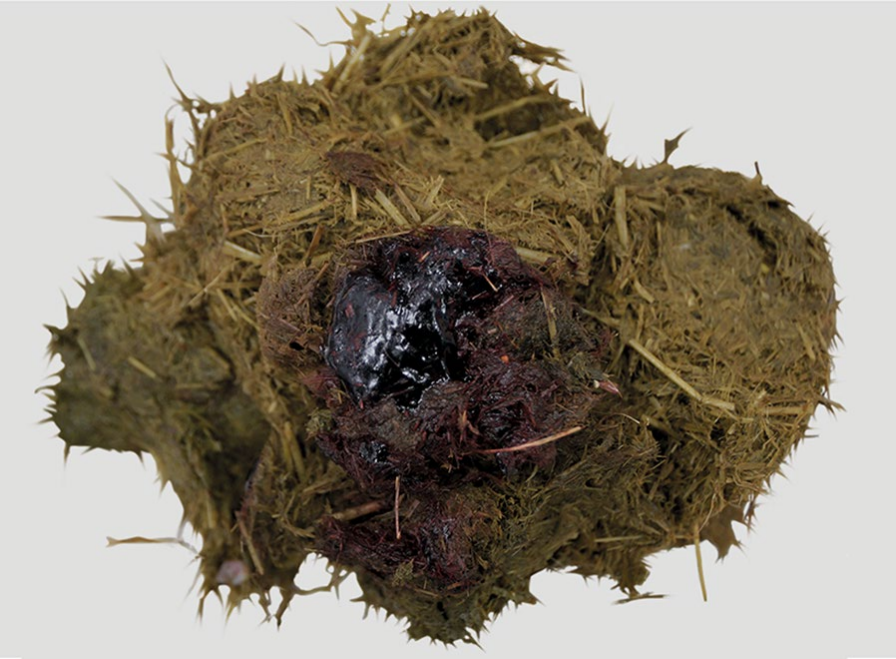
Blood clot on rumen contents present at the site of rumen perforation (cow 2). The blood clot resulted from the injury and partially sealed the rumen perforation

Suppurative fibrinous peritonitis was diagnosed, and because of the severity of lesions, both cows were euthanased and necropsied. A partially-healed scar, 4 cm in length, was seen in the left ventral abdomen approximately 25 cm cranial to the udder in cow 1. The muscle layers underneath the scar were necrotic and lacerated up to the rumen wall, which had a perforation of 3 cm in diameter (Fig. 5). The rumen wall ventral and lateral to the perforation was covered with feed particles. Yellowish fibrino-purulent exudates were evident cranial to the perforation. The peritoneal cavity was filled with yellow, turbid, foul-smelling fluid mixed with feed. In cow 2, the skin and abdominal wall of the last intercostal space at mid-level was perforated. The traumatised area was thickened, necrotic and emphysematous. There was a matching perforation in the rumen wall (Fig. 6) and extensive adhesions were present between the rumen and abdominal wall. A 6 × 12 cm blood clot was present in the area of the perforation (Fig. 7). The peritoneal cavity and omental bursa contained yellow fluid and fibrin but no feed particles were seen. The definitive diagnosis was peritonitis attributable to rumen perforation.

Hypothermia, tachycardia, enophthalmus and reduced skin turgor were indications of shock, which was most likely the result of severe inflammation caused by rumen contents in the abdomen and bacterial infection in both cows. The clinical findings were similar to those reported in cows with peritonitis localised in the left abdominal cavity and associated with rumenotomy, caesarian section and rumen trocarisation. Distension of the left flank, as reported previously, was not seen in the two cows of the present study [5]. Cow 2 had an obvious perforating skin lesion and cow 1 had multiple skin lesions suggesting that she had been injured by one or more cows. Compared to the number of reports on humans injured while in the presence of cows as well as during bull fighting [6–11], there are few descriptions of horn injuries in cattle. Implantation of a synthetic mesh has been described for repair of abdominal wall ruptures caused by horn injuries in cattle but without involvement of internal organs [12]. In three of four cows with perforating head wounds, a horn injury from another cow was thought to be the cause [13]. Horn injuries are common during transport of horned cattle [14] and bruising occurs more often among horned slaughter cattle compared with cattle without horns. Injuries may occur at sale barns and during loading, shipping, unloading and penning before the cattle are slaughtered [15].

Ultrasonography of the left flank and caudal intercostal spaces revealed inflammatory lesions of varying severity between the rumen and left abdominal wall in both cows. The accumulated fluid had displaced the rumen medially, and the lesions appeared similar to those seen in cows with peritonitis associated with rumenotomy or rumen trocarisation [5]. A tentative diagnosis of trauma was easily made after seeing the abdominal wall lesions on ultrasonograms in cow 2. In cattle with fluid accumulation between the rumen and left abdominal wall the differential diagnosis also should include complications of trocarisation, surgery, severe reticuloperitonitis, omental bursitis and perforating abomasal ulcer. Left displacement of the abomasum should be ruled out when a structure is seen between the rumen and left abdominal wall [16]. However, for the experienced clinician, differentiation of peritonitis and abomasal displacement is not difficult.

The results of haematological analysis aided in determining the severity of the illness. Severe haemoconcentration in both cows was indicative of shock, and leukopenia with a left shift was a reflection of neutrophil demand in the peritoneal cavity that overwhelmed the production capacity of the bone marrow. Hypoproteinaemia in cow 1 was attributable to loss of protein into the peritoneal cavity in association with peritonitis. Both cows had anorexia, which resulted in hypokalaemia and hypophosphataemia because of inadequate dietary intake.

## Conclusions

This case report confirms that ultrasonography is an ideal tool for characterisation of lesions located between the rumen and left abdominal wall and aids in the objective interpretation of clinical findings. Horn injury should be part of the differential diagnosis in cattle with skin wounds and severe localised peritonitis.
